# Supplementary material for: Effects of Cow’s Milk Processing on MicroRNA Levels
Source: Foods. 2023 Aug 4;12(15):2950. doi: 10.3390/foods12152950 (PMC10419269; doi:10.3390/foods12152950)
Supplement: Supplementary file 1 [file foods-12-02950-s001.zip › foods-2517438-supplementary.pdf]

foods

Supplementary Materials:

Table S1. Characteristics of the sampled farms.

Dairy farm characteristic	Number of cows	Average milk production	Grazing	Grass silage	Straw	Vetch	Alfalfa	Corn silage	Concentrate
		L/cow /day	h/day	kg fresh matter /cow/ day					
1	41	21.00	Yes	5.00	0.00	3.00	0.00	0.00	6.00
2	38	23.00	Yes	0.00	0.00	1.00	1.00	0.00	7.00
3	35	15.00	Yes	0.00	0.00	0.00	0.00	0.00	4.50
4	61	25.00	Yes	5.00	0.00	0.00	0.00	0.00	10.00
5	14	25.00	Yes	5.00	0.00	0.00	0.00	0.00	9.00
6	240	36.00	No	10.00	0.50	2.00	2.00	16.00	12.00
7	60	32.00	No	20.00	0.00	1.50	0.00	20.00	10.00
8	75	34.00	No	8.00	0.00	0.00	2.00	25.00	11.50
9	50	30.00	No	6.00	1.00	0.00	2.00	20.00	7.00
10	44	34.00	No	10.00	0.00	1.50	0.00	20.00	10.00

L/cow /day: liter per cow per day, h/day: hour per day, kg fresh matter /cow/ day: kilogram of fresh matter per cow per day

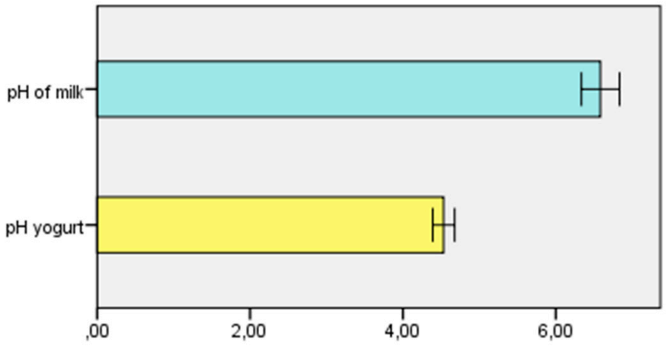

Figure S1. Mean pH and standard deviation for milk and yogurt.

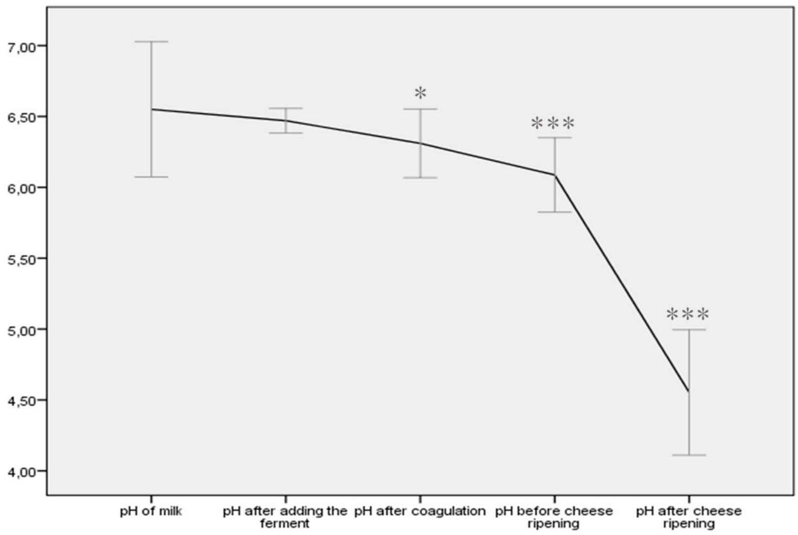

Figure S2. Evolution of pH in milk, during cheese manufacture and ripening. *P < 0.05, **P < 0.01, ***P < 0.001
